# Computer-assisted, minimally invasive transforaminal lumbar interbody fusion: One surgeon's learning curve A STROBE-compliant article

**DOI:** 10.1097/MD.0000000000011423

**Published:** 2018-07-06

**Authors:** Yun-Feng Xu, Xiao-Feng Le, Wei Tian, Bo Liu, Qin Li, Gui-Lin Zhang, Ya-Jun Liu, Qiang Yuan, Da He, Jian-Ping Mao, Bin Xiao, Zhao Lang, Xiao-Guang Han, Pei-Hao Jin

**Affiliations:** Department of Spine Surgery, Beijing Jishuitan Hospital, Beijing, China.

**Keywords:** CAMISS, computer-assisted, learning curve, minimally invasive surgery, transforaminal lumbar interbody fusion

## Abstract

Minimally invasive (MI) transforaminal lumbar interbody fusion (TLIF) is a challenging technique with a long learning curve. We combined computer-assisted navigation and MI TLIF (CAMISS TLIF) to treat lumbar degenerative disease. This study aimed to evaluate the learning curve associated with computer-assisted navigation MI spine surgery (CAMISS) and TLIF for the surgical treatment of lumbar degenerative disease. Seventy four consecutive patients with lumbar degenerative disease underwent CAMISS TLIF between March 2011 and May 2015; all surgeries were performed by a single surgeon. According to the plateau of the asymptote, the initial 25 patients constituted the early group and the remaining patients comprised the latter group. The clinical evaluation data included operative times, anesthesia times, intraoperative blood losses, days until ambulation, postoperative hospital stays, visual analog scale (VAS) leg and back pain scores, Oswestry disability index (ODI) values, Macnab outcome scale scores, complications, radiological outcomes, and rates of conversion to open surgery. The complexity of the cases increased over the series, but the complication rate decreased (12.00%–6.12%). There were significant differences between the early and late groups with respect to the average surgical times and durations of anesthesia, but no differences in intraoperative blood losses, days until ambulation, postoperative hospital stays, complication rate, VAS, ODI, Macnab outcome scale scores, or solid fusion rates. There was no need for conversion to open procedures in either group. Our study showed that a plateau asymptote for CAMISS TLIF was reached after 25 operations. The later patients experienced shorter operative times and anesthesia durations.

## 1 Introduction

Minimally invasive (MI) transforaminal lumbar interbody fusion (TLIF) is gaining popularity as a spine surgery because of its potential for minimizing soft-tissue damage and reducing blood loss, postoperative pain, and recovery time.^[1,2]^ Although MI TLIF is a technically demanding and challenging operation,^[3]^ this limited view may lead to anatomic disorientation, even for experienced surgeons,^[4]^ resulting in more hardware-associated complications than are experienced during open TLIF.^[5,6]^

Computer-assisted navigation provides excellent visualization of 3-dimensional (3D) anatomic structure relationships. Virtually any surgical instrument can be tracked on the computer monitor, in real time, in relation to the displayed anatomy.^[7]^ This advantage facilitates the complex procedures and improves the safety of the surgery. We combined the use of computer-assisted navigation techniques and MI spine surgery (CAMISS) to treat degenerative lumbar disease after hypothesizing that computer-assisted navigation would smooth the learning curve of MI TLIF and decrease its complications. To our knowledge, other literature reports have not focused on the learning curve of CAMISS TLIF. Thus, this study analyzed and quantified the learning curve associated with CAMISS TLIF, based on a single senior surgeon's experience, during the safe integration of the technique into practice.

## 2 Materials and methods

### 2.1 Patients

After obtaining institutional review board approval (Beijing Jishuitan Hospital, Beijing, China) and patient written informed consent, we conducted a retrospective evaluation of a single surgeon's first 74 patients undergoing CAMISS TLIF for symptomatic degenerative lumbar disease. The surgeon had 8 years of experience with open TLIF and 5 years of experience with computer-assisted navigation. The indications for CAMISS TLIF were spinal stenosis, spondylolisthesis (grades 1 and 2), degenerated collapsed discs, and lumbar instability, presenting in conjunction with radicular pain that was refractory to at least 6 months of medical therapy. Patients were excluded if they had spinal infections, were revision cases, or had undergone multilevel procedures. As a result, 74 patients, having undergone the procedure between March 2011 and May 2015, were included in the study.

After discharge from the hospital, patients were followed regularly (at 3 and 12 months after surgery, and annually, thereafter) and closely monitored for complications. Plain lumbar spine radiographs and, if indicated, magnetic resonance imaging or computed tomography (CT) were used to assess and confirm complications. If patients did not return for scheduled visits, follow-up was conducted through personal telephone calls.

### 2.2 Surgical technique

Each patient received general anesthesia and was positioned, prone, on a radiolucent Jackson table. Single-level CAMISS TLIF procedures were carried out through tubular retractors, as previous description.^[8]^

### 2.3 Outcome analysis

The patient records were sequentially arranged in date order. There was a subtle decrease in the operative time variability occurring at approximately patient 25. Therefore, a separate analysis was performed to compare the first 25 (early) cases with the remaining (late) cases. Thus, the first 25 consecutive patients were compared with the second 49 consecutive patients, based on their perioperative parameters, including patient demographics, operative times (minute), anesthesia times (minute), estimated intraoperative blood losses (mL), times to ambulation (days), and lengths of postoperative hospitalization (days). In addition, complications and conversion rates were compared between the 2 study groups.

The patients’ clinical results were assessed based on visual analog scores (VAS) for back and leg pain and the Oswestry disability index (ODI, version 2.0). Macnab's criteria were used to characterize the patients’ identifiable comprehensive outcomes.^[9]^ Both static and dynamic plain lumbar radiographs, taken 24 months postsurgery, were used to assess fusion; CT was performed, if necessary. The fusion grading criteria were based on the Bridwell interbody fusion grading system,^[10]^ with the assessments being performed by 2 independent assessors; a third assessor was available for adjudication.

### 2.4 Statistical analysis

All data were prospectively collected and retrospectively reviewed. SPSS, version 17.0, statistical software (SPSS, Chicago, IL) was used for the analysis. The change in operative durations over the course of the study period was evaluated using a logarithmic curve-fit regression analysis. Student's *t* test was used to compare continuous variables, the Kolmogorov–Smirnov *Z* test was used to compare nonparametric continuous variables, and the Chi-square and Fisher exact tests were used to evaluate differences in categorical variables, between the early and later cases. For all analyses, a *P* value <.05 was considered significant.

## 3 Results

**Figure 1:**
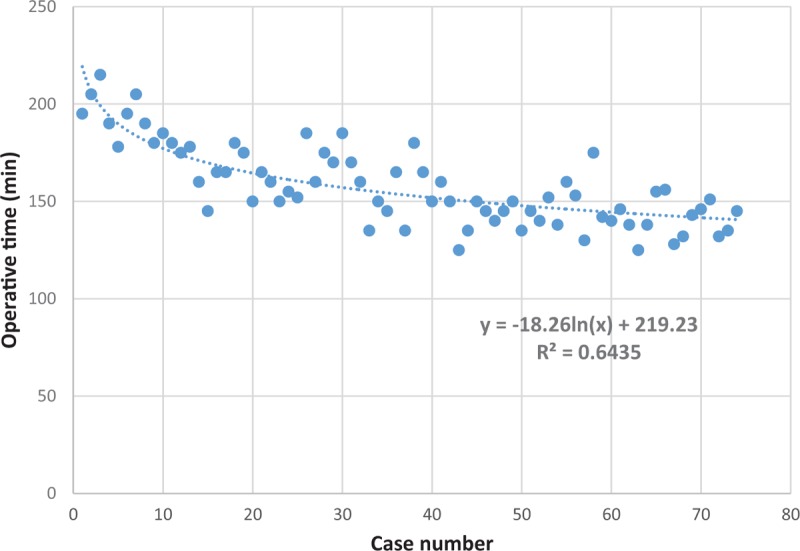
Scatterplot of the learning curve: the curve depicts 1 surgeon's results, based on total operative times. The figure represents the total time from skin incision to skin closure for the initial 74 cases. As the number of cases increased, the operative time decreased as a result of improved.

Comparing the first 25 patients undergoing single-level CAMISS TLIF to the second group of 49, there were no significant differences in patient preoperative factors (age, sex, body mass index, operation level) except pre-operative diagnoses; all the spondylolisthesis cases were in the late group (Table 1). The average operative time for the early group was 175.72 minutes. This time decreased to 149.18 minutes for the second group of 49 cases. The operative time showed a significant decrease (*P* <.001) as the case number increased, as indicated by the equation y = −18.26 ln(x) + 219.23 (x, case number; y, operative time [minute]), with a coefficient of determination *R*^2^ = 0.6435 (Fig. 1). Steady state, defined as the asymptote of the learning curve, was hypothesized to have been achieved at case number 25 (Fig. 1). There was a progressive reduction in the length of surgery over the span of the 74 cases.

**Table 1:**
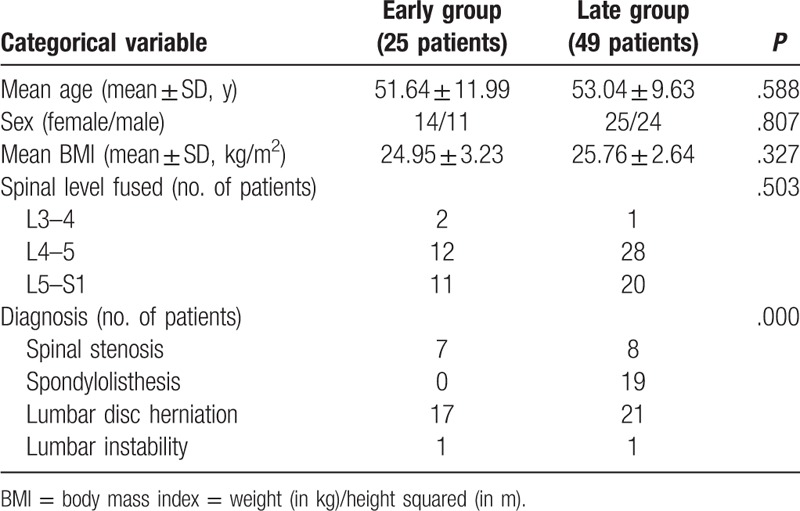
Baseline demographic characteristics and preoperative factors of the study patients.

Over the course of the study, there was a progressive increase in the difficulty of the cases undergoing the CAMISS TLIF technique, including case 30, which involved disc calcification and cases 38 and 58 that involved severe osteoporosis and the use of bone cement to strengthen the pedicle screw placement. However, the early patients required significantly longer operative times and anesthesia durations than did the later patients (both, *P* < 0.001). There were no statistical differences in intraoperative blood losses, ambulation recovery times, or postoperative hospitalization times between the 2 groups (Table 2).

**Table 2:**
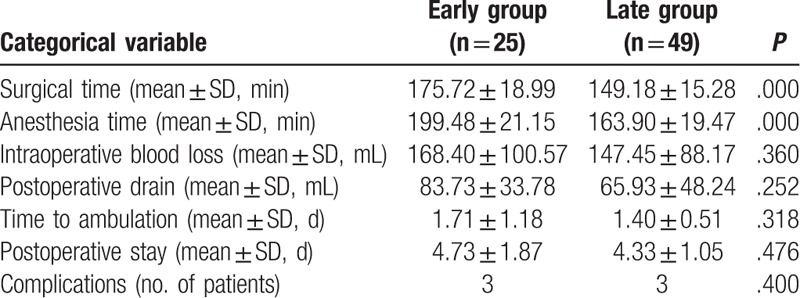
Perioperative results and outcomes.

In this cohort study, a high percentage of patient follow-up was achieved. At 24 months, 90.54% of patients returned for their follow-up visit (7 patients were too distant from the hospital to return for a follow-up; they were followed-up by telephone and sent their 24-month radiology reports to the hospital). Both groups exhibited similar pre-operative baseline pain and disability scores, and all patients demonstrated postoperative clinical improvement. An analysis of the back and leg pain VAS scores revealed improved postoperative scores; there were no significant differences between the groups at any follow-up point. Both groups showed similar improvements in ODI scores at 3, 12, and 24 months after surgery, compared with their preoperative scores; there was no significant difference in the ODI scores between the 2 groups at the any follow-up point (Table 3). In assessing the Macnab scale, the outcomes were excellent/good in 84.00% and 87.76% of patients in the early and late groups, respectively (*P* = 0.920) (Table 4).

**Table 3:**
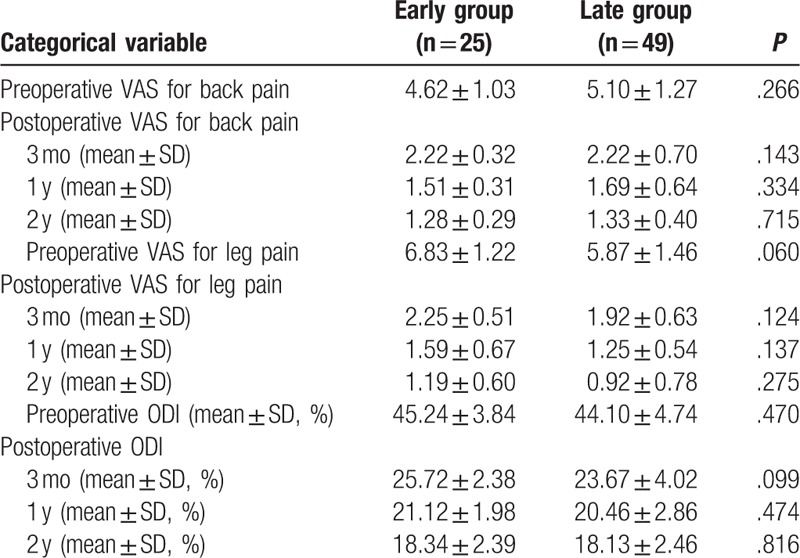
Visual analog scale (VAS) scores for back and leg pain, and Oswestry disability index (ODI) scores.

**Table 4:**
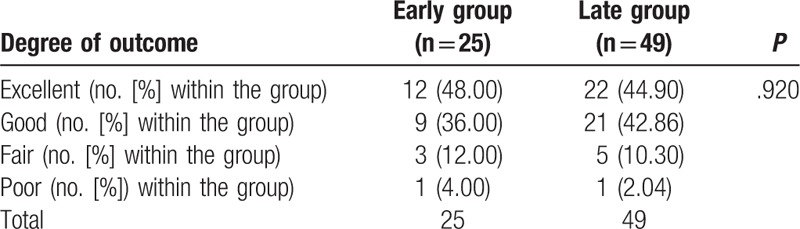
Macnab criteria outcomes.

Among the 74 patients, none of the CAMISS TLIF cases required conversion to open surgery, and only 6 patients (8.11%) experienced complications. In the early group, patient 13 suffered right, L5 root palsy due to a local hematoma requiring emergent reoperation; the patient completely recovering within 3 months. At the most recent follow-up, this patient's weakness had resolved and she was classified as having a “good outcome”. Patients 5 and 18 suffered intraoperative dural tears during nerve decompression. In the late group, patient 30 suffered radicular pain due to cage migration at L5–S1, after beginning ambulation; the pain disappeared after revision surgery to reinsert the cage and compress the adjacent vertebrae through the previous incision. Patient 38 suffered a dural tear, had a calcified disc, and severe spinal canal stenosis, and was a complex case with a long operative time. The patient developed a cerebrospinal fluid leak from the axilla of the nerve root during dissection. The 3 dural tears were small, and the overlying fascia was tightly closed, without additional exposure or repair. No specific treatment was prescribed, postoperatively, for the cerebrospinal fluid leak condition other than 6 hours of bed rest. The drain was driven out a week postoperative, and a deep stich was given for drain osculum, without any neurological sequelae or wound complications. Patient 41 suffered pseudoarthros is with few symptoms and rejected additional invasive treatment. Pedicle screw-related complications were not observed. The overall complication rate was 12.00% for the early group and 6.12% for the late group; this difference was not significant (*P* = .400).

Fusion status was judged based on the 24-month follow-up radiographs. According to Bridwell's criterion, there were 13 grade I cases, 8 grade II cases, and 4 grade III cases in the early group; the late group demonstrated 23 grade I cases, 17 grade II cases, 8 grade III cases, and 1 grade IV case. The rates of “good” fusions (grades I and II) were 84.00% in the early group and 81.63% in the late group (*P* = .964).

## 4 Discussion

MI TLIF is a technically challenging operation that requires complex spinal procedures in a limited working space.^[3]^ Frequent criticisms of MI spine surgery procedures include its steep learning curve^[11]^ and the increased likelihood of complications developing while surgeons are gaining experience with these new techniques.^[12]^

A limited number of publications have addressed the MI TLIF learning curve. Lee et al^[3]^ assessed 1 senior surgeon achieved proficiency after 44 surgeries during his first 90 consecutive cases of single-level MI TLIF. Nandyala et al^[13]^ reported their first 65 cases of single-level MI TLIF and found the technically difficult surgery to have a high complication rate (30.77%). Another group^[14]^ described 1 surgeon's first 86 cases of MI TLIF, a plateau asymptote was reached after 30 cases, and noted a steep learning curve in the initial cases.

In this study, we reviewed 1 surgeon's initial 74 single-level CAMISS TLIF procedures and found that he reached a stable operative time after 25 cases. In the present study, as the surgeon's experience increased, the operative times were reduced, and the number of complex cases increased, including the inclusion of patients with spondylolisthesis and severe osteoporosis patients who required cement to strengthen pedicle screw placement in the late group. Among the late group of CAMISS TLIF patients, the average procedure time was 26 minutes faster than in the early group. The reduction in operative time may have been related to the improvement in the surgeon's skill, as well as that of the first assistant, scrub nurses, and fluoroscopic technician.^[3]^ With the surgeon's experience increasing, the scope of eligible patients was broadened, and more complex cases were included.

An assessment of surgical complications is useful for improving the safety and quality of treatment. In our study, the total complication rate was 8.11% (6/74), which was lower than previously reported rates of 30.77%^[13]^ and 10.47%.^[14]^ These other groups found that the main technical complications included pedicle screw malpositioning, cage migration, and dural tears. Therefore, the key steps for MI TLIF success include accurate pedicle screw insertion and adequate neurological decompression. Weinstein et al^[15]^ reported frequent false-positive and false-negative results when only biplanar fluoroscopy was use to assess the screw placement. Lee et al^[14]^ showed that 2 of their initial 86 cases required revision due to pedicle screw misplacement during MI TLIF, and Nandyala et al^[13]^ similarly reported this complication in 1 of their initial 33 cases. Furthermore, during MI TLIF decompression, the field cannot be directly visualized, nor the structures touched, as is possible during open TLIF. Visualization through the tubular retractor limits the surgeon's ability to check the decompression area, making the procedure more difficult and inaccurate, and increasing the risk that some structure might be missed, resulting in inadequate decompression.^[12]^

The advent of new instrumentation has helped to improve the efficiency of the surgical procedure and reduced the number of iatrogenic complications. Intraoperative 3D navigation provides real-time visualization of the complex spinal structures, facilitates preoperative planning, and provides intraoperative guidance.^[7]^ During pedicle insertion, 3D navigation can display the real-time, 3D structure, and guide the surgeon's choice of entry point, to improve the safety and efficiency of the trajectory.^[16]^ Navigation also facilitates nerve decompression, allowing better visualization of the targeted segment, assisting in accurately docking the tubular retractor, and ensuring the scope of decompression scope by avoiding anatomic disorientation. If necessary, a postoperative scan can also be performed, before closing, to ensure sufficient decompression and evaluate the implant's position. With navigational assistance, the relative positioning of the tool and the bone may be accomplished in real-time, further enhancing the surgeon's confidence in completing a complex operation.

The incidence of complications between the early and late groups, in this study, was not significantly different (*P* = 0.400), despite more complex cases being involved in the late group of patients than in the early group. The lack of difference may be influenced by 2 factors. First, the early group involved less complex surgeries than the late group, suggesting that the surgeon's increased experience compensated for the increased procedural complexity in the late group. Second, the surgeon may have paid more attention during the early procedures, prolonging the operative duration and reducing the rate of complications. Increased experience brought increased efficiency and shorter operative times, but did not produce increased complications, consistent with previous reports.^[17,18]^ As surgeons become more skilled with this new procedure, their confidence in the procedure increases, improving their ability to cope with more complex cases and allowing further exploration of the system's versatility.

Durotomy is a potential risk during decompression procedures. The surgeon needs to be a master of spinal anatomy to discern the thecal sac and nerve roots through the limited working channel. We resected the ligament flava, after completing the ostectomy, to reduce the risk of durotomy. Fortunately, the dural tears we encountered were small, and the overlying fascia was tightly closed without additional exposure or repair. When the retractors were removed, the muscles closely approximated over the surgical bed, creating a physical barrier to prevent hydrostatic pressure from driving cerebrospinal fluid flow into this newly created space.^[19]^ We experienced 1 epidural hematoma that could have been avoided had more careful attention been given to bipolar cauterization of the epidural veins. For cage migration, 2 lessons were learned from this early experience. First, a cage of sufficient size should be selected to restore the disc height and open neural foramen, compressing adjacent vertebral bodies to restructure the natural lordosis, before tightening the fixation nuts. Second, fluoroscopy should be used to confirm that the cage is optimally positioned, before closing.

One of the study's limitations is that the surgeon performed other MI spinal procedures during the study period, and these additional microsurgical surgeries may have helped improve his CAMISS TLIF operative proficiency. Second, the operator had previously mastered the navigation skills, which may have introduced some bias into the present research.

## 5 Conclusion

Our study provided experimental proof of our hypothesis that computer-assisted navigation enhances a surgeon's ability to accurately and safely perform complex MI spinal procedures. The results indicated that a plateau asymptote of CAMISS TLIF was reached after 25 cases, with later patients requiring shorter operative and anesthesia times. Computer-assisted navigation and suitable patient selection can help shorten the learning curve and decrease the complication rate; after the initial stage of the learning curve, the scope of eligible candidates may be broadened.

## Author contributions

**Conceptualization:** Yunfeng Xu, Wei Tian.

**Data curation:** Yunfeng Xu, Xiaofeng Le, Bin Xiao, Peihao Jin.

**Formal analysis:** Yunfeng Xu, Xiaofeng Le, Zhao Lang, Xiaoguang Han.

**Funding acquisition:** Wei Tian, Yunfeng Xu.

**Investigation:** Yunfeng Xu, Xiaofeng Le.

**Methodology:** Wei Tian, Bo Liu, Liu, Qiang Yuan, Da He, Jian-Ping Mao.

**Project administration:** Wei Tian, Bo Liu, Yajun Liu, Qiang Yuan, Da He.

**Resources:** Wei Tian, Qiang Yuan, Da He.

**Software:** Xiaofeng Le, Bin Xiao.

**Supervision:** Wei Tian, Qin Li, Guilin Zhang, Yajun Liu, Qiang Yuan, Da He, Jian-Ping Mao.

**Validation:** Wei Tian, Bo Liu, Qin Li, Guilin Zhang, Yajun Liu, Qiang Yuan, Da He, Jian-Ping Mao.

**Visualization:** Zhao Lang, Xiaoguang Han, Peihao Jin.

**Writing – original draft:** Yunfeng Xu, Xiaofeng Le.

**Writing – review & editing:** Yunfeng Xu.
